# Jejunal GIST: Hunting down an unusual cause of gastrointestinal bleed using double balloon enteroscopy. A case report

**DOI:** 10.1016/j.ijscr.2019.06.053

**Published:** 2019-06-27

**Authors:** Diana Mellisa Dualim, Guo Hou Loo, Reynu Rajan, Nik Ritza Kosai Nik Mahmood

**Affiliations:** Department of General Surgery, Faculty of Medicine, The National University of Malaysia, Jalan Yaacob Latiff, Bandar Tun Razak, Postcode 56000, Selangor, Malaysia

**Keywords:** Laparoscopic, Obscure GI bleeding, Double-balloon enteroscopy, Small bowel bleeding, Gastrointestinal stromal tumour, Capsule endoscopy

## Abstract

**Introduction:**

Gastrointestinal stromal tumours (GISTs) are the most common mesenchymal neoplasms of the alimentary tract but accounts for only 0.1–3% of all gastrointestinal neoplasms. The most common presentation of GISTs is acute or chronic gastrointestinal bleeding, in which the patient presents with symptomatic anaemia.

**Presentation of case:**

With that in mind, we describe a 66-year-old man who presented with recurrent episodes of obscure gastrointestinal bleeding for two years. Video capsule endoscopy (VCE) showed several small telangiectasias in the proximal small bowel. Oral route double-balloon enteroscopy (DBE) revealed abnormal mucosa 165 cm from incisor with central ulceration and vascular component. He subsequently underwent surgical excision. The histopathological report confirmed the diagnosis of GIST arising from the jejunum. During his clinic follow up, he remains symptom-free with no evidence of recurrence.

**Discussion:**

The diagnosis of bleeding small intestine GISTs can be challenging as these are inaccessible by conventional endoscopy. Imaging modalities such as double-balloon enteroscopy, capsule endoscopy, CT angiography, intravenous contrast-enhanced multidetector row CT (MDCT) and magnetic resonance enterography (MRE) have been used to assist in the diagnosis of bleeding small intestine GISTs. The mainstay of management for small intestine GIST is complete surgical excision.

**Conclusion:**

Bleeding jejunal GIST is very rare and only a handful of case reports have been published. The mainstay of management for small intestine GIST is complete surgical excision. It is essential to obtain a complete excision of localised disease and avoiding tumour spillage in order to reduce the risk of local recurrence and metastatic spread of GISTs.

## Highlights

• Bleeding jejunal GIST is very rare with only a handful of published case reports.
• Double-balloon enteroscopy and capsule endoscopy can be used to diagnose bleeding small intestine GIST.
• Occult small bowel bleeding can go undetected for years.
• Age is one of the determining factors for the type of small bowel pathology detected.
• Surgical resection remains the mainstay treatment for GIST.
• Laparoscopic surgery offers similar oncologic outcomes as an open surgery.

## 1 Introduction

Small intestinal bleeding is a rare presentation with a prevalence of 5–10% in patients presenting with gastrointestinal bleeding [6]. A patient presented with overt bleeding can be evaluated first with an upper and lower endoscopy to exclude upper and lower bleeding that can be readily reached with a standard endoscope [6]. GIST arising from the jejunum is a very rare cause of small intestinal bleeding [5]. The diagnosis of bleeding small intestine GISTs can be challenging as these are inaccessible by conventional endoscopy [4]. This case has been reported in line with the SCARE criteria [11].

## 2 Case presentation

A 66-year-old gentleman with no known comorbidities presented to us with a history of multiple melenic bowel movements. He has associated lethargy and easy fatiguability as well. Further history from the patient revealed that he had been treated for symptomatic anaemia for the past two years. On clinical examination, he was pale, tachycardic but normotensive. His abdominal examination was unremarkable and digital rectal examination revealed melena. Initial investigations revealed a drop of haemoglobin from 11 g/dl to 4 g/dl. He was promptly resuscitated with blood products, and an early upper endoscopy was performed.

The index oesophagogastroduodenoscopy (OGDS) showed a small Forrest 3 antral ulcer with multiple subcentimeter gastric polyps [13]. The gastric polyps were biopsied, and the histopathology subsequently reveals it to be benign. A colonoscopy showed blood-stained colonic mucosa in its entirety. However, no bleeding source was identified. An urgent contrast-enhanced computed tomography (CECT) of the abdomen was performed which revealed no significant abnormality. There were no bowel related masses seen. After the acute gastrointestinal bleeding episode subsided, we proceeded to work him up with a presumptive diagnosis of possible small intestinal bleed.

**Fig. 1:**
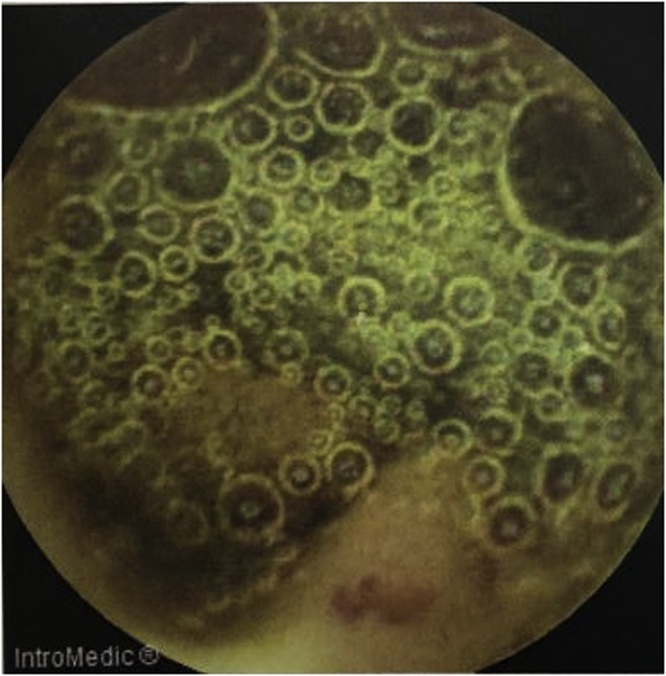
Still image from Video Capsule Endoscopy showing a small telangiectasia over the proximal part of small bowel.

**Fig. 2:**
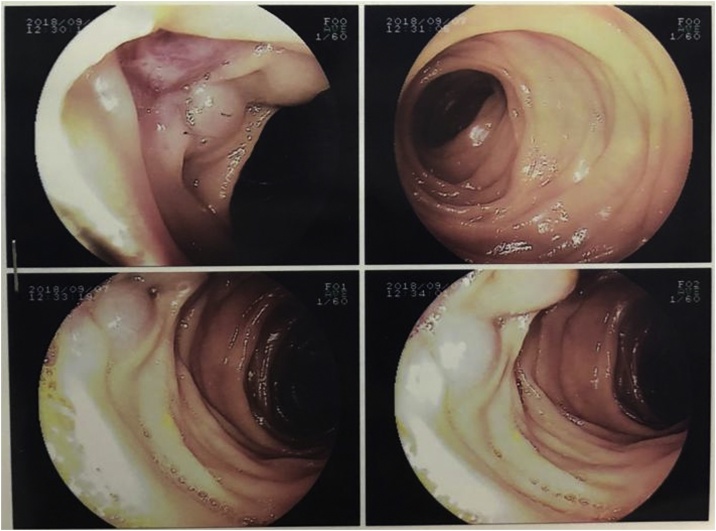
Images from a double-balloon enteroscopy showing abnormal vascularity with a central umbilication over the mucosa of the small bowel, located 165 cm from the incisor.

**Fig. 3:**
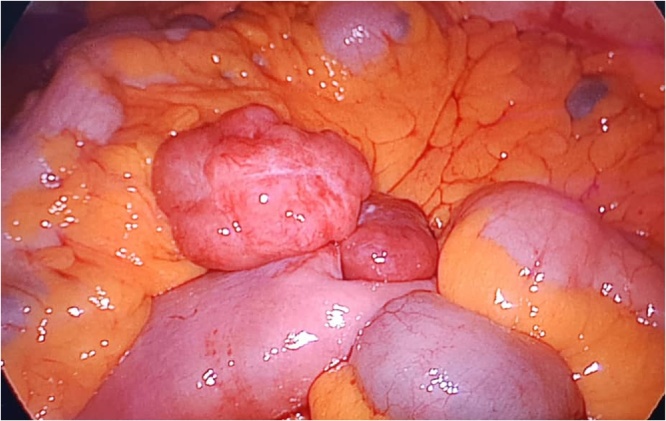
Intraoperative image from laparoscopic camera showing an exophytic lesion measuring 6 cm × 6 cm × 3 cm approximately 30 cm distal to the duodenojejunal flexure.

**Fig. 4:**
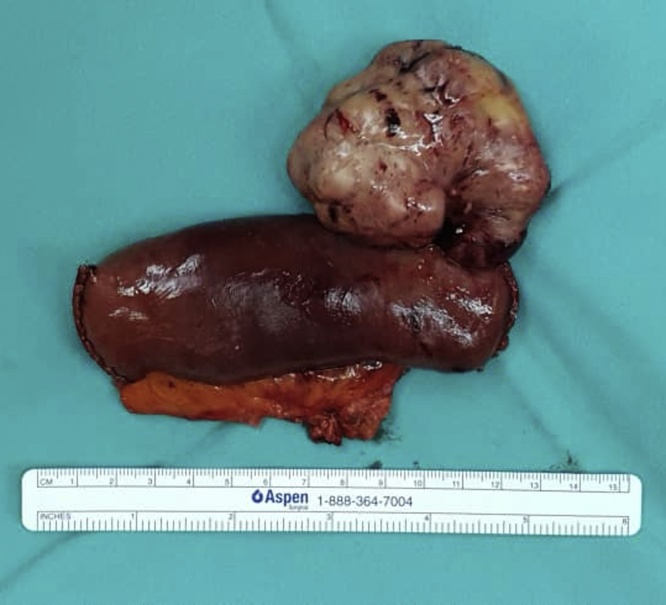
Resected gross specimen of jejunal GIST, measuring 6 cm × 6 cm × 3 cm.

**Fig. 5:**
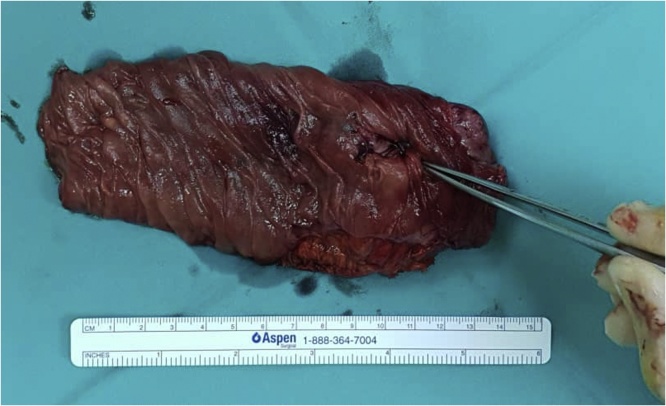
Dissected small bowel (jejunum) specimen with GIST. Central umbilication is clearly seen (shown using forceps).

Capsule endoscopy was performed which showed several small telangiectasias in the proximal part of the small bowel (Fig. 1). There was no visible tumour, polyps or ulcers. A double-balloon enteroscopy was then performed. It showed abnormal vascularity with a central umbilication over the mucosa of the small bowel (Fig. 2). The mucosal abnormality was located at 165 cm from the incisor. Preoperative assessment of the patient showed he has an ASA score of 1 and a Revised Cardiac Risk Index score of 1 [12]. He subsequently underwent a laparoscopic examination in the theatre. Laparoscopic entry was performed with a closed technique (Veress needle) [14]. Intraoperative findings revealed an exophytic lesion measuring 6 cm × 6 cm × 3 cm approximately 30 cm distal to the duodenojejunal flexure (Fig. 3, Fig. 4, Fig. 5). Small bowel resection and primary side-to-side anastomosis were performed, without regional lymphadenectomy. He recovered uneventfully and was discharged after a short hospital stay.

The histopathological report confirmed the diagnosis of GIST arising from the jejunum, with a TNM staging of T3N0M0. The tumour measures 60 × 65 × 30 mm, with a mitosis of 1 per 50 high power field (HPF), with no evidence of tumour rupture. On immunohistochemistry staining, the tumour is positive for CD117 and DOG1. During his clinic follow up, he remains symptom-free with no evidence of recurrence or metastases at six months follow up.

## 3 Discussion

Small intestinal bleeding is a rare presentation with a prevalence of 5–10% in patients presenting with gastrointestinal bleeding [6]. Source of bleeding is from the ampulla of Vater to the ileocaecal valve. Multiple investigation tools are available to diagnose small intestinal bleeding including capsule endoscopy (CE), deep enteroscopy, angiography and enterography examination [6]. Since the introduction of capsule endoscopy and deep enteroscopy, the majority of patient classified as having obscure gastrointestinal bleeding were identified to have bleeding from the small intestine [7].

There are many etiologies which can cause small bowel bleeding. Age is one of the determining factors for the type of small bowel pathology detected [6]. According to Gerson et al., the source of bleeding in patients under the age of 40 years is usually Meckel’s diverticulum or inflammatory bowel disease [6]. For patients over the age of 40 years, vascular lesions such as angioectasia, and ulcers secondary to anti-inflammatory agents are more common. It is important to note that small bowel angioectasia are the most common cause of small bowel bleeding [6].

Small intestinal bleeding usually presents as stable overt or an occult bleed. A patient presented with haematemesis, melena or haematochezia is termed overt bleeding whereas an asymptomatic patient with faecal occult blood test positive is termed occult bleeding [6]. A patient presented with overt bleeding can be evaluated first with an upper and lower endoscopy to exclude upper and lower bleeding that can be readily reached with a standard endoscope. The terminal ileum should be intubated during colonoscopy to examine the ileal mucosa and to exclude bleeding source from a more proximal lesion [6]. Upper endoscopy in our patient revealed a small antral ulcer with multiple small gastric polyps without stigmata of recent haemorrhage whereas his colonoscopy does not reveal any significant abnormality.

Capsule endoscopy (CE) is the recommended first-line procedure for small bowel evaluation after upper and lower gastrointestinal bleeding source have been excluded [6]. It is usually performed before deep enteroscopy. Deep enteroscopy can be considered as the initial diagnostic tool if CE is contraindicated or in cases of massive haemorrhage [6]. When there is a strong suspicion of small bowel bleeding with abnormal VCE study, it should be followed by total deep enteroscopy [6]. Double-balloon enteroscopy is one of the methods of deep enteroscopy.

Gastrointestinal stromal tumours (GISTs) are the most common mesenchymal neoplasms of the alimentary tract but accounts for only 0.1–3% of all gastrointestinal neoplasms [1,2]. The most common sites of origin for GISTs are the stomach (60%–70%), the small intestine (25%–35%), the oesophagus (2%–3%), and rarely in the colon, the rectum or the appendix (5%) [3]. It is known to originate from the smooth muscle pacemaker interstitial cell of Cajal which has a function of coordinating gut motility and peristalsis [8].

The symptoms of GISTs are nonspecific and depend on the size and location. Small GISTs (<2 cm) are usually found incidentally from endoscopy or imaging for other pathology as many of them are asymptomatic. The most common symptom is gastrointestinal bleeding, which is present in 50% of the patients, followed by abdominal pain (20–50%) and gastrointestinal obstruction (10–30%) [3,8]. Other symptoms include melena, hematemesis, fullness, and a palpable mass. They rarely spread to the regional lymph node or other extra-abdominal organs, but they can metastasise to the liver [3,8].

According to the latest NCCN guidelines, a computed tomography (CT) scan of the abdomen is the initial workup for the evaluation, staging, and monitoring of treatment response. GIST on contrast-enhanced CT shows the characteristic of a well-defined soft tissue of relatively low density, which is homogenous [8]. In our patient, however, the CT of the abdomen failed to reveal any significant abnormalities. From the endoscopic view, GISTs form a well-delineated spherical or hemispheric mass, arising mostly from the muscularis propria layer beneath the mucosa and pushing it to the lumen to form a smoothly contoured elevation [8].

Surgical resection remains the mainstay treatment for non-metastatic GISTs [1,8]. Size of less than 2 cm with no signs of malignancy can be managed with active surveillance unless it is symptomatic then surgical resection is needed. However, a small tumour size does not exclude the malignant potential in a GIST [8]. Laparoscopic surgery has the advantage earlier recovery, less painful and has better cosmetic results. Furthermore, oncologic outcomes between laparoscopic and open surgery are similar [9,10]. As submucosal and lymphatic invasion is rare in GIST, this permits local excision rather than formal organ resection. Wide margins and lymph node dissections are not necessary. This allows laparoscopic resection to be one of the alternative techniques compared to more invasive open surgery [10].

The Asian consensus guidelines recommend laparoscopic resection for GIST up to 5 cm size when it is located in a favourable location [9]. The principles of surgical resection are obtaining a negative margin to ensure complete excision of localised disease and maintaining an intact capsule to prevent tumour spillage and subsequent trocar seeding [10]. A negative gross surgical margin is a pertinent factor in decreasing the risk of local recurrence and metastatic spread of GIST. Positive margins should be removed at the primary resection [10]. According to the Asian consensus guidelines, endoscopic resection is not suitable for small GIST due to the risk of damaging the pseudo-capsule, which may increase the risk of recurrence [9].

There are four independent prognostic factors for recurrence after complete surgery. These factors include the tumour size (cm), mitosis per 50 high power field (HPF), tumour location (gastric versus non-gastric) and tumour rupture [9]. Amongst these factors, tumour rupture is the worst prognostic factor as most ruptured GISTs have recurrences during follow-up [9]. Mitosis index also is one of the factors which is strongly related to recurrence. Mitotic rate and tumour size are the two main factors that are independent but mutually inﬂuential predictors for metastases [9].

## 4 Conclusion

GIST arising from the jejunum is a very rare cause of small intestinal bleeding. The diagnosis of bleeding small intestine GISTs can be challenging as these are inaccessible by conventional endoscopy. Normal findings from an upper and lower endoscopy in a patient presenting with gastrointestinal bleeding symptoms should raise a suspicion of small intestinal bleed. Imaging modalities such as double balloon enteroscopy, capsule endoscopy, CT angiography, intravenous contrast-enhanced multidetector row CT (MDCT) and magnetic resonance enterography (MRE) may be used to assist in the diagnosis of bleeding small intestine GISTs. The mainstay of management for small intestine GIST is complete surgical excision. It is essential to obtain a complete excision of localised disease and avoiding tumour spillage in order to reduce the risk of local recurrence and metastatic spread of GISTs.

## Funding

No source of funding.

## Ethical approval

The National University of Malaysia’s Ethics Committee has exempted the need for an ethical approval for any case report being written/published.

## Consent

Written informed consent was obtained from the patient for publication of this case report and accompanying images.

## Author contribution

Study concepts: Nik Ritza Kosai, Reynu Rajan, Study design: Nik Ritza Kosai, Reynu Rajan.

Data acquisition: Diana Mellisa Dualim, Loo Guo Hou, Quality control of data and algorithms: Nik Ritza Kosai, Reynu Rajan, Data analysis and interpretation: Diana Mellisa Dualim, Loo Guo Hou, Statistical analysis: -Not applicable.

Manuscript preparation: Loo Guo Hou, Diana Mellisa Dualim.

Manuscript editing: Loo Guo Hou.

Manuscript review: Nik Ritza Kosai, Reynu Rajan.

## Registration of research studies

Not applicable.

## Guarantor

Professor Dr Nik Ritza Kosai.

## Provenance and peer review

Not commissioned, externally peer-reviewed.

## Declaration of Competing Interest

No conflict of interests.
